# Global prevalence and mortality of severe *Plasmodium malariae* infection: a systematic review and meta-analysis

**DOI:** 10.1186/s12936-020-03344-z

**Published:** 2020-07-31

**Authors:** Manas Kotepui, Kwuntida Uthaisar Kotepui, Giovanni D. Milanez, Frederick R. Masangkay

**Affiliations:** 1 Medical Technology, School of Allied Health Sciences, Walailak University, Tha Sala, Nakhon Si Thammarat, Thailand; 2 Department of Medical Technology, Far Eastern University, Manila, Philippines

**Keywords:** Severe, Complications, *P. malariae*, Quartan malaria

## Abstract

**Background:**

Severe complications among patients with *Plasmodium malariae* infection are rare. This is the first systematic review and meta-analysis demonstrating the global prevalence and mortality of severe *P. malariae* infection in humans.

**Methods:**

The systematic review and meta-analysis followed the Preferred Reporting Items for Systematic Reviews and Meta-Analyses (PRISMA) guidelines. All research articles published on the severity and mortality of *P. malariae* infection cases in humans were retrieved from three public databases: PubMed, Scopus, and ISI Web of Science. The pooled prevalence estimate and 95% confidence interval (CI) of complications in patients with *P. malariae* malaria was analysed using the random-effects model provided in Stata software. The pooled odds ratio (OR) and 95% CI of severe malaria for *P. malariae* infection and *Plasmodium falciparum* infection were analysed using Review Manager software.

**Results:**

Six studies were used to estimate the pooled prevalence of severe *P. malariae* malaria. Out of 10,520 patients infected with *P. malariae*, the pooled prevalence estimate of severe *P. malariae* infection was 3% (95% CI 2–5%), with high heterogeneity (I^2^: 90.7%). Severe anaemia (3.32%), pulmonary complications (0.46%), and renal impairments (0.24%) were the most common severe complications found in patients with *P. malariae* infection. The pooled proportion of severe anaemia for *P. malariae* infection and *P. falciparum* infection was comparable among the four included studies (OR: 0.74, 95% CI 0.22–2.45, I^2^ = 98%). The pooled proportion of pulmonary complications was comparable between patients with *P. malariae* infection and those with *P. falciparum* infection among the four included studies (OR: 1.44; 95% CI 0.17–12.31, I^2^: 92%). For renal complications, the funnel plot showed that the pooled proportion of renal complications for *P. malariae* infection and *P. falciparum* infection was comparable among the four included studies (OR: 0.94, 95% CI 0.18–4.93, I^2^: 91%). The mortality rate of patients with *P. malariae* infection was 0.17% (18/10,502 cases).

**Conclusions:**

This systematic review demonstrated that approximately two percent of patients with *P. malariae* infection developed severe complications, with a low mortality rate. Severe anaemia, pulmonary involvement, and renal impairment were the most common complications found in patients with *P. malariae* infection. Although a low prevalence and low mortality of *P. malariae* infection have been reported, patients with *P. malariae* infection need to be investigated for severe anaemia and, if present, treated aggressively to prevent anaemia-related death.

## Background

Malaria is a public health problem, with an estimated 219 million cases in 87 countries, and 435,000 malaria-related deaths were estimated in 2017 [1]. The highest prevalence is in the World Health Organization (WHO) African Region [1]. There are five major *Plasmodium* species which can infect human beings, including *Plasmodium falciparum*, *Plasmodium vivax*, *Plasmodium malariae*, *Plasmodium ovale*, and *Plasmodium knowlesi* [2]. The transmission of malaria in humans occurs through the bite of a female *Anopheles* mosquito [2]. In addition, blood transfusion and maternal transmission have been demonstrated as other routes of malaria infection with less frequent occurrences [3].

Most malaria diseases in humans are caused by *P. falciparum* and *P. vivax* [4]. Both species were geographically present in the southeastern and western Pacific regions, with a higher distribution of *P. falciparum* than *P. vivax* in Africa, while *P. vivax* is more prevalent than *P. falciparum* in South America [5]. *P. ovale* infection is widespread principally in tropical Africa, whereas *P. knowlesi* infection occurs only in certain forested areas of Southeast Asian countries [5]. *P. malariae* is frequently co-endemic with *P. falciparum* in sub-Saharan Africa, South America, Indonesia, Southeast Asia, and the western Pacific [6]. A previous study found that 7.4% of *P. malariae* infections were mixed infections with *P. falciparum* [7].

Severe malaria is any complication of *Plasmodium* species infection that develops rapidly and leads to death within hours or days [8], and these complications were listed by the WHO [9]. Most severe malaria-related deaths are caused by *P. falciparum*, while *P. vivax* or *P. ovale* can also induce severe complications resulting in death but less frequently than *P. falciparum* [10]. Severe malaria is a rare complication that occurs in patients with *P. malariae* infection [11], including acute kidney injury [12–15], cerebral malaria [16], and prostrated and multiple convulsions [17]. This is the first systematic review and meta-analysis to demonstrate the prevalence of severe *P. malariae* infection among patients using articles published in three research databases.

## Methods

### Literature search

The systematic review and meta-analysis followed the Preferred Reporting Items for Systematic Reviews and Meta-Analyses (PRISMA) guidelines [18]. All research articles published on the prevalence of severe *P. malariae* cases in humans were retrieved from three public databases: PubMed, Scopus, and ISI Web of Science. The published articles were searched up to 31 December 2019. The search terms were “(severe OR complicated OR Complication) AND (“Plasmodium malariae” OR malariae)” using Boolean operators “OR” or “AND”. EndNote software version X7 (Thomson Reuters, New York, NY) was used to process all references in the present study.

### Eligibility criteria

The inclusion criteria were any study that reported severe *P. malariae* infection and any primary study published in the English language. Only articles reporting severe *P. malariae* mono-infection were included. Those included studies were used to analyse the pooled prevalence of severe *P. malariae* malaria. Malaria diagnosis was based on one of the following methods: microscopy, rapid diagnostic test (RDT), and polymerase chain reaction (PCR).

Exclusion criteria were any study reporting *P. malariae* infection with no related topics/no complications, animal studies, drug studies, case reports and case series, editorials, letters, proceedings, short communications, experimental studies, malaria in pregnancy, co-infection with *P. malariae* and other *Plasmodium* species or other diseases, and review articles. Studies were also excluded if they reported incomplete data on severe *P. malariae* infection.

### Data selection and extraction

Two reviewers (MK and KUK) selected the article independently. Any discrepancy between two reviewers about article selection was resolved by discussion or by requesting the opinion of the third reviewer (GDM). The data set of this systematic review and meta-analysis, including the name of the author(s), publication year, study period, age distribution, haemoglobin concentration, parasitaemia level, nephrotic syndrome, albuminuria, the number of all malaria cases for each species, mortality rate, and the number of severe cases of all *Plasmodium* species, were extracted into Microsoft Excel spreadsheets (Microsoft Corporation, USA) for further analysis.

### Criteria of severe malaria

The criteria of severe *P. malariae* malaria were based on the WHO definition among the included studies [8].

### Quality of included studies

A quality assessment was performed following the quality assessment tool developed by the Newcastle–Ottawa Scale (NOS) for assessing the quality of nonrandomized studies in meta-analyses [19]. The quality assessment tool was used to evaluate the validity of the included studies, which is also shown in Table 3.

### Statistical analysis and meta-analysis

Qualitative variables, including the number of patients with severity signs, the number of deaths, and the number of patients infected with *Plasmodium* spp., were analysed using the frequency and percentage. Data in Microsoft Excel spreadsheets were transferred into Stata version 14 (StataCorp, USA) for calculating the pooled prevalence and 95% confidence interval (CI) of complications in patients with severe *P. malariae* malaria. The pooled prevalence estimate of severe *P. malariae* infection was calculated using the number of patients with severe malaria and the number of patients with *P. malariae* malaria. The pooled proportion of severe complications for *P. malariae* infection was calculated using the number of severe complications among patients with *P. malariae*, and the total number of severe complications among patients infected with all *Plasmodium* spp., which have been reported in the included studies. The estimation of the pooled prevalence was following a previous research guideline [20]. The command in Stata was “metaprop case population, random”. Moreover, the summary odds ratio (OR) and 95% CI were calculated to present the difference in the proportion of severe *P. malariae* infection compared with non-*malariae* infection and were also determined using Review Manager (RevMan) (Cochrane Collaboration, UK). The degree of heterogeneity between the included studies was assessed using Cochran’s Q test and Moran’s I^2^ (inconsistency), with I^2^ values of more than 50% indicating substantial heterogeneity.

### Publication bias

Funnel plot analysis was used to demonstrate any symmetry in the funnel plots among patients with *P. malariae* infection and to evaluate whether publication bias was the underlying cause of data heterogeneity in the included studies.

## Results

### Characteristics of included studies

**Fig. 1:**
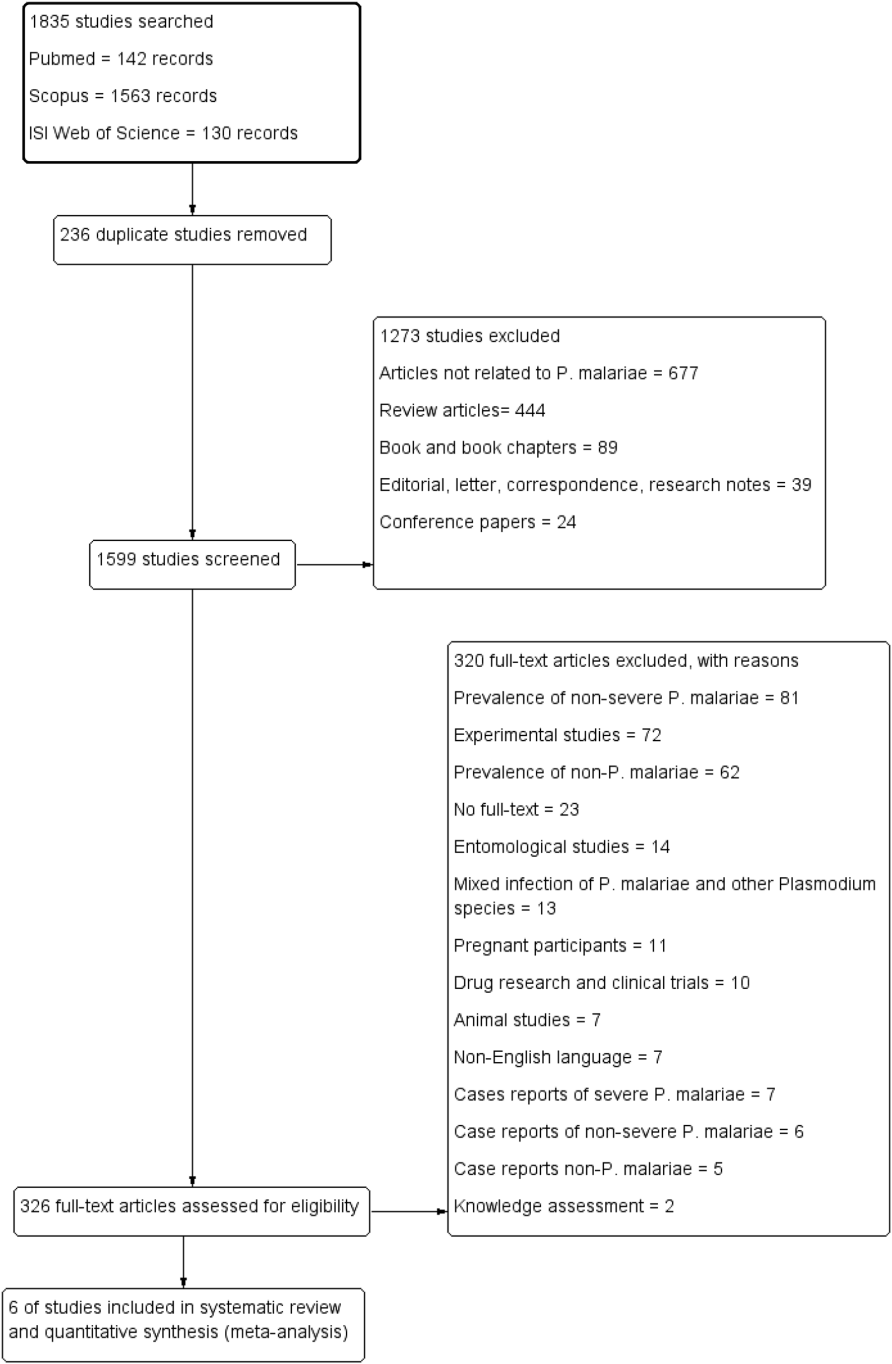
Flowchart for study selection

After searching for possible related studies, a total of 1835 studies were retrieved. Among those 1835 studies, 236 duplicate studies were removed. Then, 1599 studies were screened by title and abstracts, and 1273 studies were excluded, including 677 articles not related to *P. malariae*, 89 book and book chapters, 24 conference papers, 39 editorials/letters/correspondence articles, 39 note articles, and 444 review articles. The full texts of a total of 326 articles were screened for eligibility, and 320 were excluded, including 81 non-severe *P. malariae*, 72 experimental studies, 62 non-*P. malariae*, 23 no full-text, 14 entomological studies, 13 mixed infection of *P. malariae* with other *Plasmodium* species, 11 Pregnant participants, 10 drug research and clinical trials, 7 animal studies, 7 non-English language, 7 case reports of severe *P. malariae*, 6 case reports of non-severe *P. malariae*, 5 case reports of non-*P. malariae*, and 2 knowledge assessment. Six articles were included in the final qualitative and quantitative analysis (Fig. 1). All characteristics of the included studies are demonstrated in Table 1. Among the six included studies, five studies were observational cross-sectional designs in which data were retrieved from hospitals [21, 22] or national medical records [23–25]. One study was a prospective cohort study conducted in Belgium during 2000–2005 [26]. In the present systematic review and meta-analysis, two included studies were conducted in Belgium and Sweden, European countries [25, 26]; two were conducted in Indonesia, an Asian region [21, 22]; one in the USA, North America [24]; and one in Colombia, South America [23]. Data on the haemoglobin concentration (g/dl), nephrotic syndrome, albuminuria, age distribution, and parasitaemia level are shown in Additional file 1.

**Table 1:** Characteristics of the included studies

No	Reference	Study area (years of the survey)	Research design	All *Plasmodium* spp.	Severe malaria	Deaths	Impaired consciousness	Severe anaemia	Renal impairment	Hyperbilirubinaemia	Pulmonary complications	Shock
1.	Bottieau et al. [26]	Belgium (2000–2005)	Prospective cohort study	*P. vivax *= 48 *P. ovale *= 34 *P. malariae *= 16	*P. vivax *= 8 *P. malariae *= 3 *P. ovale *= 4					*P. vivax *= 8 *P. ovale *= 4 *P. malariae* = 3		
2.	Chaparro et al. [23]	Colombia (2010)	Medical records	*P. vivax *= 82,856 *P. falciparum *= 32,777 *P. malariae *= 47 Mixed = 1428	*P. falciparum *= 282 *P. vivax *= 293 *P. malariae *= 6 Mixed = 32		*P. vivax *= 44 *P. falciparum *= 59 *P. malariae *= 1		*P. vivax *= 66 *P. falciparum *= 88 *P. malariae *= 2	*P. vivax *= 130 *P. falciparum *= 99 *P. malariae *= 1	*P. vivax *= 34 *P. falciparum *= 31 *P. malariae *= 2	
3.	Douglas et al. [21]	Indonesia (2004–2012)	Medical records	*P. falciparum *= 44,171 *P. vivax *= 28,841 *P. ovale *= 110 *P. malariae *= 4045 Mixed = 14,206	*P. falciparum *= 2444 *P. vivax *= 1050 *P. malariae *= 95 *P. ovale *= 0 Mixed = 718			*P. falciparum *= 2466 *P. vivax *= 1050 *P. ovale *= 0 *P. malariae *= 95 Mixed = 718				
4.	Hwang et al. [24]	USA (1985–2011)	Medical records	*P. falciparum *= 15,272 *P. vivax *= 12,152 *P. ovale *= 903 *P. malariae *= 1254 Mixed = 226	*P. falciparum *= 1416 *P. vivax *= 163 *P. malariae *= 22 *P. ovale *= 18 Mixed = No report	*P. falciparum *= 122 *P. vivax *= 10 *P. ovale *= 2 *P. malariae *= 2	*P. falciparum *= 514 *P. vivax *= 37 *P. ovale *= 4 *P. malariae *= 2	*P. falciparum *= 140 *P. vivax *= 16 *P. ovale *= 6 *P. malariae *= 3	*P. falciparum *= 503 *P. vivax *= 48 *P. ovale *= 6 *P. malariae *= 7		*P. falciparum *= 176 *P. vivax *= 35 *P. ovale *= 4 *P. malariae *= 2	
5.	Langford et al. [22]	Indonesia (2004–2013)	Medical records	*P. falciparum *= 100,078 *P. vivax *= 65,306 *P. ovale *= 120 *P. malariae *= 5097 Mixed = 25,779	*P. falciparum *= No report *P. vivax *= No report *P. malariae *= 232 *P. ovale *= No report Mixed = No report	*P. malariae *= 16		*P. falciparum *= 2521 *P. vivax *= 1099 *P. ovale *= 0 *P. malariae *= 250 Mixed = 782	*P. falciparum *= 415 *P. vivax *= 81 *P. ovale *= 0 *P. malariae *= 16 Mixed = 84		*P. falciparum *= 1095 *P. vivax *= 938 *P. ovale *= 1 *P. malariae *= 44 Mixed = 343	
6.	Wangdahl et al. [25]	Sweden (1995–2015)	Medical records	*P. falciparum *= 1548 *P. vivax *= 776 *P. ovale *= 188 *P. malariae *= 61 Mixed = 44	*P. falciparum *= 146 *P. vivax *= 60 *P. malariae *= 2 *P. ovale *= 10 Mixed = 8	*P. falciparum *= *3* *P. vivax *= *0* *P. ovale *= *0* *P. malariae *= *0* Mixed = 1	*P. falciparum *= *28* *P. vivax *= *1* *P. ovale *= 1 *P. malariae *= *0* Mixed = 1	*P. falciparum *= *16* *P. vivax *= *15* *P. ovale *= *0* *P. malariae *= 1 Mixed = 4	*P. falciparum *= *31* *P. vivax *= *1* *P. ovale *= *0* *P. malariae *= 0 Mixed = 3	*P. falciparum *= 66 *P. vivax *= *28* *P. ovale *= *6* *P. malariae *= 0 Mixed = 4	*P. falciparum *= 15 *P. vivax *= *4* *P. ovale *= *2* *P. malariae *= 0 Mixed = 0	*P. falciparum *= *33* *P. vivax *= *8* *P. ovale *= *2* *P. malariae *= *1* Mixed = 3
	Total			*P. falciparum *= 93,768 (four studies: 2, 3, 4, 6) *P. vivax *= 124,625 (five studies: 1, 2, 3, 4, 6) *P. malariae *= 10,520 (all six studies) *P. ovale *= 1235 (four studies: 1, 3, 4, 6) Mixed = 15, 678 (three studies: 2, 3, 6)	*P. falciparum *= 4, 288 (four studies: 2,3,4,6) *P. vivax *= 1574 (five studies: 1, 2, 3, 4, 6) *P. malariae *= 360 (all six studies) *P. ovale *= 32 (four studies: 1, 3, 4, 6), Mixed = 758 (three studies: 2, 3, 6)							

### The crude prevalence of severe *P. malariae* infection and severe non-*malariae* infection

All six included studies reported a total of 10,520 patients with *P. malariae* infection. The lowest number of cases of *P. malariae* infection was 16 patients in a study conducted by Bottieau et al. [25], while the highest number of cases of *P. malariae* infection was 5097 patients in a study by Langford et al. [22]. Among the 10,520 patients who were infected with *P. malariae*, three hundred and sixty (3.4%) developed severe *P. malariae* infection (Table 1). The included studies reported patients with *P. falciparum* (193,846 cases), *P. vivax* (189,931 cases), *P. ovale* (1355 cases), and mixed infection (41,457 cases). The crude severity rates of infections with other *Plasmodium* spp. were 4.57% *P. falciparum* (four studies), 1.26% *P. vivax* (five studies), 2.59% *P. ovale* (four studies), and 4.83% mixed infection. The number of severity signs of patients with *P. malariae* and other *Plasmodium* spp. infection are demonstrated in Table 2. Severe anaemia was the most common severe complication found among patients with *P. malariae* infection (349 cases, 3.32%). Severe anaemia was also the most common complication found in *P. falciparum* (3.86%), *P. vivax* (1.52%), *P. ovale* (0.66%), and mixed infection (4.73%). Pulmonary complications (0.46%) and renal impairment (0.24%) were the second and third most common complications found in patients with *P. malariae* infection, respectively.

**Table 2:** Complications presented with *P. malariae* and other *Plasmodium* spp.

Major complication (WHO, 2014)	Number of severity signs (severity rate, %)				
	*P. malariae* (N = 10,520 cases, 6 studies)	*P. falciparum* (N = 193,846 cases, 5 studies: 2, 3, 4, 5, 6)	*P. vivax* (N = 189,931 cases, 6 studies)	*P. ovale* (N = 1355 cases)	Mixed infection (N = 41,457 cases)
Acidosis	0	21 (0.01)	0	0	0
Pulmonary/lung	48 (0.46)	1317 (0.68)	1011 (0.53)	7 (0.52)	343 (0.83)
Death	18 (0.17)	122 (0.06)	10 (.0050)	2 (0.15)	1 (0.002)
Cerebral malaria	3 (0.03)	601 (0.31)	82 (0.04)	5 (0.37)	1 (0.002)
Convulsions	0	9 (0.005)	0	0	0
Renal impairment	25 (0.24)	1037 (0.53)	218 (0.11)	6 (0.44)	87 (0.21)
Prostration	0	9 (0.005)	1 (0.0005)	0	0
Hypotension/shock	1 (0.01)	33 (0.02)	8 (0.004)	2 (0.15)	3 (0.007)
Jaundice	4 (0.04)	165 (0.09)	166 (0.09)	6 (0.44)	4 (0.01)
Severe anaemia	349 (3.32)	7473 (3.86)	2886 (1.52)	9 (0.66)	1961(4.73)
Bleeding/DIC	0	17 (0.009)	5 (0.003)	1 (0.07)	1 (0.002)
Hyperparasitaemia	0	57 (0.03)	0	0	3 (0.007)
Hypoglycaemia	0	2 (0.001)	0	0	0
Total	448 (4.26)	10,842 (5.59)	4387 (2.31)	38 (2.8)	2404 (5.8)

### The pooled prevalence estimate of severe *P. malariae* malaria

**Fig. 2:**
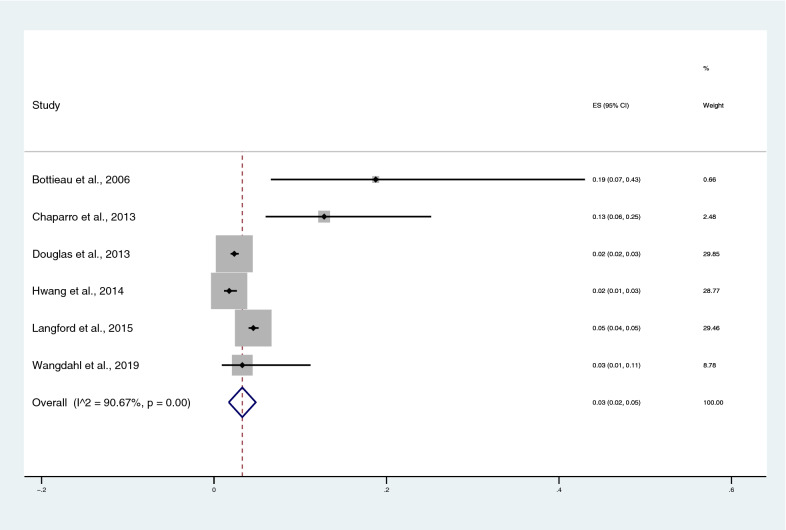
The pooled prevalence estimate of severe *P. malariae* infection

The number of patients with severe *P. malariae* infection and the total number of patients with *P. malariae* infection were used to estimate the pooled prevalence of severe *P. malariae* infection (Additional file 2). The pooled prevalence estimate of severe *P. malariae* infection was 3% (95% CI 2–5%), with high heterogeneity (I^2^: 90.67%, P < 0.001), using random-effects analysis (Fig. 2). The highest proportion of severe *P. malaria* infection was found in a study by Bottieau et al. (19%, 95% CI 7–43%) [26].

### The pooled prevalence estimate of complications for *P. malariae* malaria

**Fig. 3:**
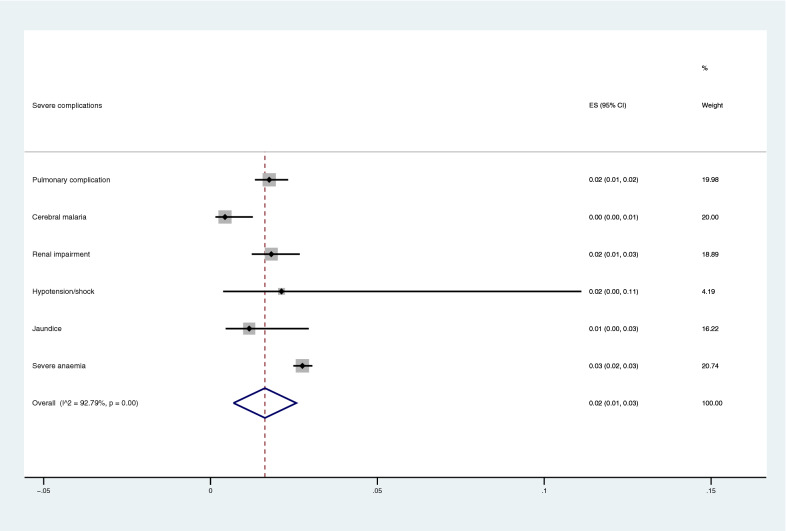
The pooled proportion of severe complications for severe *P. malariae* infection

The number of severe complications among patients with *P. malariae*, and the total number of severe complications among patients infected with all *Plasmodium* spp. were used to estimate the pooled proportion of severe complications for *P. malariae* infection in comparison to those with *Plasmodium* spp. (Additional file 3). The pooled proportion of severe complications for *P. malariae* infection in comparison to those with *Plasmodium* spp. was demonstrated in Fig. 3. Overall, the pooled proportion of severe complications in patients with *P. malariae* infection was 2% (95% CI 1–3%). The highest proportion of severe complications caused by *P. malariae* infection in comparison to other *Plasmodium* spp. was severe anaemia (3%, 95% CI 2–3%).

### The pooled proportion of severe anaemia for *P. malariae* infection and *P. falciparum* infection

**Fig. 4:**
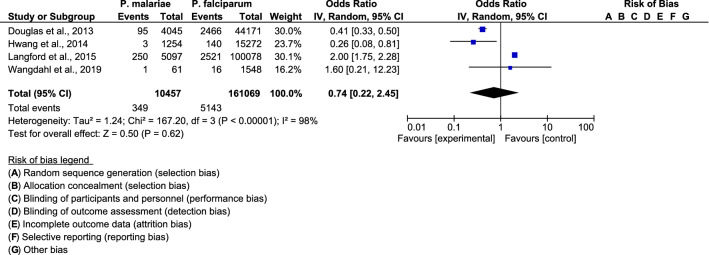
The pooled proportion of severe anaemia for *P. malariae* infection and *P. falciparum* infection

The pooled OR of severe anaemia for *P. malariae* infection and *P. falciparum* infection was comparable among the four included studies (OR: 0.74, 95% CI 0.2245–2., I^2^ = 98%) (Fig. 4). The proportion of severe anaemia in *P. malariae* infection was lower than in *P. falciparum* infection in a study by Douglas et al. (OR: 0.41, 95% CI 0.33–0.50) [21], and Hwang et al. (OR: 0.26, 95% CI 0.08–0.81) [24]. The proportion of severe anaemia in *P. malariae* infection was higher than that in *P. falciparum* infection in a study by Langford et al. (OR: 2.00, 95% CI 1.75–2.28) [22].

### The pooled proportion of pulmonary and renal complications for *P. malariae* infection and *P. falciparum* infection

**Fig. 5:**
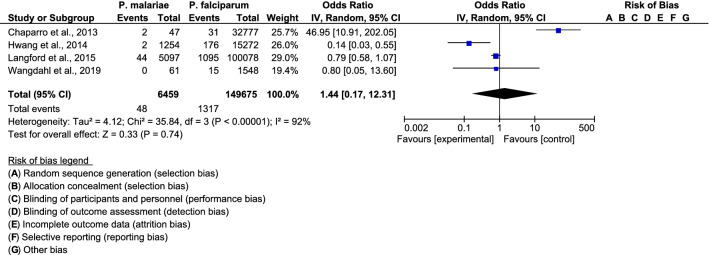
The pooled proportion of pulmonary complications for *P. malariae* infection and *P. falciparum* infection

**Fig. 6:**
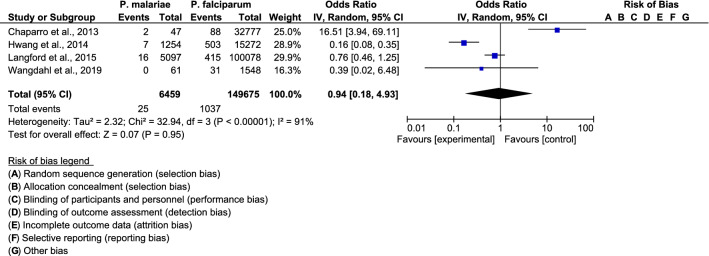
The pooled proportion of renal complications for *P. malariae* infection and *P. falciparum* infection

The pooled proportion of pulmonary complications was comparable between patients with *P. malariae* infection and those with *P. falciparum* infection among the four included studies (OR: 1.44; 95% CI 0.17–12.31, I^2^: 92%) (Fig. 5). Pulmonary complications were lower in *P. malariae* infection than in *P. falciparum* infection in a study by Hwang et al. (OR: 0.14; 95% CI 0.03–0.55) [24], while pulmonary complications were higher in *P. malariae* infection than in *P. falciparum* infection in the study by Chaparro et al. (OR: 46.95; 95% CI 10.9–202.1) [23]. For renal complications, the funnel plot showed that the pooled proportion of renal complications for *P. malariae* infection and *P. falciparum* infection was comparable among the four included studies (OR = 0.94, 95% CI 0.18–4.93, I^2^: 91%) (Fig. 6). Renal complications were lower in patients with *P. malariae* infection than in those with *P. falciparum* infection in the study by Hwang et al. (OR: 0.16; 95% CI 0.08–0.35) [24], while renal complications were higher in patients with *P. malariae* infection than in those with *P. falciparum* infection in the study by Chaparro et al. (OR: 16.51; 95% CI 3.94–69.1) [23].

### The mortality of *P. malariae* infection

The crude mortality rate of *P. malariae* infection was 0.17% (18 cases), as reported in the included studies by Hwang et al. [24], and by Langford et al. [22]. The crude mortality rates of infections with other *Plasmodium* spp. reported by the included studies were *P. falciparum* (122 cases, 0.08%), *P. vivax* (10 cases, 0.01%), *P. ovale* (2 cases, 0.15%), and 1 case of mixed infection (0.002%).

### Quality of included studies

All six studies included in the present study were rated according to the NOS guidelines. Overall, five studies were rated good, with a maximum of nine stars, and one study was rated medium with eight stars because of their “Representativeness of the cases” (presented only severe anaemia complications in their study, with no other complications in their objectives). The rating details are provided in Table 3.

**Table 3:** Quality of included studies

No.	Reference	Selection				Compatibility	Exposure		
		Is the case definition adequate?	Representativeness of the cases	Selection of controls	Definition of controls		Ascertainment of exposure	Same method of ascertainment for cases and controls	Non-response rate
1.	Bottieau et al. [26]	*	*	*	*	**	*	*	*
2.	Chaparro et al. [23]	*	*	*	*	**	*	*	*
3.	Douglas et al.[21]	*		*	*	**	*	*	*
4.	Hwang et al. [24]	*	*	*	*	**	*	*	*
5.	Langford et al. [22]	*	*	*	*	**	*	*	*
6.	Wangdahl et al. [25]	*	*	*	*	**	*	*	*

### Publication bias

The funnel plot analysis to demonstrate the publication bias among the six included studies could not be performed, as the analysis required a minimum of 10 studies [27].

## Discussion

This is the first systematic review and meta-analysis demonstrating the pooled prevalence estimate of severe *P. malariae* infection. The results demonstrated that the pooled prevalence estimate of severe *P. malariae* malaria was low (3%). These results were in accordance with a previous study indicating that severe *P. malariae* infection was a rare complication with less frequent occurrence in malarial patients [11]. The mechanism of severe malaria among *P. malariae* infections is unknown. *P. malariae* is a slow-growing parasite infecting mature red blood cells [28]. It can result in a lower number of merozoites produced per 72-h erythrocytic cycle, called quartan or tertian malaria, inducing the earlier development of human immunity [6]. *P. malariae* is not the cause of malarial relapse from persistent liver-stage parasites, but the recrudescence of blood-stage parasites can persist for long periods without signs or symptoms. Even with appropriate treatment, chronic subclinical *P. malariae* infection can occur because of its extended prepatent period when the inadequate drug in the blood cannot eliminate newly emerging merozoites [28, 29]. As most patients infected with *P. malariae* infections have no sign or symptoms of malaria, they have no need for health services during their asymptomatic infection [30]. Even though patients suspected of having *P. malariae* infections visit the health service, *P. malariae* parasites are missed by light microscopy due to their low parasite density compared to that of *P. falciparum* parasites [31, 32]. It is also difficult to distinguish *P. malariae* from other *Plasmodium* species using a microscopic method, and its prevalence may be underestimated, resulting in late disease presenting severe complications [17, 33]. In addition, *P. malariae* infection can easily be misdiagnosed when microscopy is used for detection and is often treated as a bacterial infection [12]. The detection of *P. malariae* by microscopy in patients with mixed infection with *P. falciparum* in endemic areas where *P. falciparum* predominates is also difficult, as, in the area of malaria endemicity in Africa, infections of *P. malariae* can frequently be mixed with *P. falciparum* infections [6]. Another possible cause of severe malaria in patients with *P. malariae* infection is rosette formation, i.e., the binding of two or more infected red cells to an uninfected red cell, which is associated with microcirculatory blood flow obstruction in *P. falciparum* infection and has been reported in patients with *P. malariae* infection [17].

The present systematic review and meta-analysis demonstrated that severe anaemia was the most common complication in patients with *P. malariae* infection, as reported by the included studies. The included study by Douglas et al. [21], demonstrated that the proportion of severe anaemia in *P. malariae* infection was lower than that in non-*P. malariae* infection. They indicated that *P. malariae* infection was associated a lower mean haemoglobin level than infection with other *Plasmodium* spp., [21]. The included study by Langford et al., 2015, enrolled 5097 patients with *P. malariae* infections and demonstrated that the mean haemoglobin concentration was lower (9.0 g/dl) in patients with *P. malariae* infection than in those with infection with all other *Plasmodium* species [22]. Nevertheless, the lower haemoglobin concentrations associated with a greater risk of death were reported in patients with *P. falciparum* infection [21]. This causation of anaemia in malarial patients might be due to the higher parasitaemia level of *P. falciparum* and potentially cause a rapid drop in haemoglobin [21]. Previous studies suggested that the severe anaemia during *P. malariae* infection might be due to prolonged erythrocyte destruction and bone marrow suppression with a minimal reduction of erythrocyte lifespan by a low parasitaemia level [21, 22, 34]. It should be noted that most patients with *P. malariae* infection in the included studies were Highland Papuans, who are the ethnic group with the highest risk of severe anaemia caused by nutritional and haematological factors [22]. These factors can potentially contribute to the lower mean haemoglobin level in these patients, as the risk of severe anaemia in *P. malariae* infections was similar to that for *P. falciparum* and *P. vivax* infections after adjusting for ethnicity in the multivariable model [22].

Regarding the age distribution of patients with *P. malariae* infection, the included study by Langford et al., clarified that most patients with *P. malariae* infections were aged more than 15 years, which was older than those with *P. falciparum*, *P. vivax,* and mixed infections, reflecting the low transmission intensity in Indonesia [22]. A low number of patients with *P. malariae* infections were aged less than 5 years (10.9%), the most vulnerable group affected by malaria [35]. This systematic review and meta-analysis indicated that pulmonary complications and renal impairment were frequently found in patients with *P. malariae* infection. The mechanism of pulmonary complications in patients with severe *P. malariae* infection is unknown. Lung injury in *P. malariae* malaria might be caused by microvascular sequestration, similar to *P. falciparum* infection, which can increase vascular permeability, resulting in fluid loading and progression to pulmonary oedema [36]. Nevertheless, further study is needed to investigate whether red blood cells parasitized with *P. malariae* cytoadhere to endothelial cells of humans.

Renal impairment and nephrotic syndrome were commonly present in patients with *P. malariae* infection, with a mixed IgM and IgG immune complex located at the renal basement membrane [37]. *P. malariae* infection has long been recognized as the causative agent of nephrotic syndrome among untreated *P. malariae* infections [29, 38–40]. In addition, albuminuria was commonly seen in patients treated with *P. malariae* for neurosyphilis in the 1930s [41]. Nevertheless, the included study by Langford et al. demonstrated a very low prevalence of nephrotic syndrome (0.1%) among 5097 enrolled patients with *P. malariae* infections [22]. Although a lower prevalence of nephrotic syndrome occurred in *P. malariae* infections, the proportion of nephrotic syndrome was higher in *P. malariae* infection than in infections with the other *Plasmodium* species [22]. Previous studies demonstrated that *P. malariae*-induced nephrotic syndrome can lead to progressive renal failure, particularly in young adults [42, 43]. *P. malariae*-induced acute renal failure after treatment with quinine was reported previously. In this case, treatment with intravenous quinine and four rounds of renal dialysis improved the renal function of the patient [12]. An unusual case of transfusion-transmitted *P. malariae* infection in an individual with thalassemia major remained undiagnosed for several months, and the individual eventually developed acute renal failure [15].

Severe malaria caused by *P. malariae* infection is infrequent and is caused by multiple susceptibility genes: genes related to inflammation, including tumour necrosis factor (TNF), interleukin-6 (IL-6) and IL-10, macrophage migration inhibitory factor (MIF), angiotensin-converting enzyme (ACE), and catalase; genes for coagulation factors, including plasminogen activator inhibitor (PAI)-1; fibrinogen; coagulation factors II, V, VII, and XIII; and genes related to innate immunity, including toll-like receptor (TLR)-2, TLR-4, TLR-5, mannose-binding lectin (MBL), interleukin 1 receptor associated kinase 1 (IRAK-1), cluster of differentiation-14 (CD-14), toll interleukin-1 receptor-associated protein (TIRAP), and Nf-κB inhibitor (IκB) [13]. In addition, a previous study indicated that five important genes, including IRAK-1 rs1059703, CD-14 rs2569190, TNF-beta rs909253, IL-6 rs1800795, and MIF rs755622, were associated with the increased severity of multiple organ dysfunction syndrome in a case of *P. malariae* infection [44].

The present systematic review and meta-analysis had several limitations. First, the number of included studies was limited, which might affect the pooled estimate of severe *P. vivax* malaria prevalence globally. Second, although severe *P. malariae* infection was associated with a high burden of severe anaemia, the quantitative analysis of haemoglobin concentrations in *P. malariae* infection could not be performed, as it was reported quantitatively only in the included study by Douglas et al. [21]. Third, nephrotic syndrome and albuminuria could not be analysed, as only the included study by Langford et al. [22] reported the number of *P. malariae* infections with nephrotic syndrome. Fourth, the age distribution of patients with *P. malariae* infections could not be analysed, as only the included study by Langford et al. reported the age distribution of patients with *P. malariae* infections. Fifth, the parasitaemia level related to severe *P. malariae* infection could not be analysed, as only the included study by Bottieau et al. [26] reported the parasitaemia level. In view of all results, although the present study reported a low prevalence of severe *P. malariae* infection, those with *P. malariae* infection need to be investigated for anaemia and, if present, treated aggressively to prevent anaemia-related death.

## Conclusion

This systematic review demonstrated the low prevalence and low mortality of severe malaria among patients with *P. malariae* infection. Severe anaemia, pulmonary complications, and renal impairment were the common complications found in patients with *P. malariae* infection. Despite the low prevalence and mortality of *P. malariae* infection, infected patients need to be investigated for severe anaemia and, if present, treated aggressively to prevent anaemia-related death.

## Supplementary information

**Additional file 1.** Demographic and laboratory data.

**Additional file 2.** Data for calculate the pooled prevalence estimate of severe *P. malariae* infection.

**Additional file 3.** Data for calculate the pooled prevalence estimate of complications for *P. malariae* malaria.

## Data Availability

The data sets used during the current study are available in the present manuscript and supplementary files.

## Abbreviations

PRISMA: Preferred Reporting Items for Systematic Reviews and Meta-Analyses
CI: Confidence interval
OR: Odds ratio
WHO: World Health Organization
RDT: Rapid diagnostic test
PCR: Polymerase chain reaction
NOS: Newcastle–Ottawa Scale
TNF: Tumour necrosis factor
IL: Interleukin
MIF: Macrophage migration inhibitory factor
ACE: Angiotensin-converting enzyme
PAI: Plasminogen activator inhibitor
TLR: Toll-like receptor
MBL: Mannose-binding lectin
IRAK-1: Interleukin 1 receptor associated kinase 1
CD: Cluster of differentiation
TIRAP: Toll interleukin-1 receptor-associated protein
IκB: Nf-κB inhibitor
