# Supplementary material for: Global prevalence and mortality of severe Plasmodium malariae infection: a systematic review and meta-analysis
Source: Malar J. 2020 Jul 31;19:274. doi: 10.1186/s12936-020-03344-z (PMC7395392; doi:10.1186/s12936-020-03344-z)
Supplement: Supplementary file 1 — Additional file 1. Demographic and laboratory data. [file 12936_2020_3344_MOESM1_ESM.docx]

**Hemoglobin, nephrotic syndrome, albuminuria, age distribution, and parasitemia levels in the six included studies**

| No | Reference | Hemoglobin (g/dl) | Nephrotic syndrome | Albuminuria | Age distribution | Parasitemia level |
| --- | --- | --- | --- | --- | --- | --- |
| 1. | Bottieau et al., 2006 | Not shown | Not shown | Not shown | 35 (29–53) | Parasitemia < 500/µL, 14/27 (52%): Geometric mean parasitemia/µL, 479 (76–3,020) |
| 2. | Chaparro et al., 2013 | Not shown | Not shown | Not shown | Not shown | Not shown |
| 3. | Douglas et al., 2013 | 8.93±2.54 | Not shown | Not shown | Not shown | Not shown |
| 4. | Hwang et al., 2014 | Not shown | Not shown | Not shown | Not shown | Not shown |
| 5. | Langford et al., 2015 | Not shown | 4 (0.1%) | Not shown | 0 to <1 (80, 1.6%)  1 to <5 (554, 10.9%),  5 to <15 (1,096, 27.5%)  > 15 (3,367, 66.1%) | Not shown |
| 6. | Wangdahl et al., 2019 | Not shown | Not shown | Not shown | 30.2 (3–65) | Not shown |
